# Epidemic Cerebro-Spinal Meningitis: Vaccine Treatment

**Published:** 1907-08-31

**Authors:** 


### EPIDEMIC CEREBRO-SPINAL MENINGITIS:

### Vaccine Treatment.

A most remarkable recovery from cerebro-spinal meningitis hitherto observed has been recorded by a group of Liverpool observers—Rundle, Mottram, Orr, and Williams.* In their case the specific organism was isolated from the cerebro-spinal fluid, a vaccine was prepared from this organism and inoculated at intervals varying from five days up to eight days, five inoculations being given in all. The result was brilliant; the child, who had all the evidences of progressive cerebro-spinal meningitis, and who, prior to the inoculations, was going steadily from bad to worse, made a complete recovery. Improvement did not manifest itself until forty-eight hours after the first inoculation. Then the rigidity began to relax. After each inoculation the temperature fell to normal, and remained down for increasing periods after successive inoculations, the other signs of the disease meanwhile diminishing and ultimately clearing up completely. The mode of preparation of the vaccine is described by the authors as follows: "A twenty-four hours old culture was inoculated into sterile normal saline solution and placed in a water-bath at 60° C. for half an hour; agar tubes were inoculated from it, and incubated over-night to test sterility. The number of cocci contained was estimated by mixing known volumes of washed normal blood and vaccine; these were then diluted with normal saline solution, films prepared at once and stained by Leishman's method. The number of organisms present was counted against the red blood-corpuscles. The vaccine was freshly prepared for each of the first four doses; the fifth had been made seven days before use. No local reaction was observed after any of the injections." On each occasion the opsonic index was found to rise after the inoculation, and generally to a very remarkable extent.

* Lancet, July 27, 1907, p. 220.

